# Negative Affect Influences Electrophysiological Markers of Visual Working Memory in Mildly Stressed Older Adults

**DOI:** 10.3389/fnagi.2018.00148

**Published:** 2018-05-22

**Authors:** Tab R. Memmott, Daniel Klee, Barry Oken

**Affiliations:** Oken Cognitive Neuroscience Laboratory, Department of Neurology, Oregon Health and Science University (OHSU), Portland, OR, United States

**Keywords:** negative affect, visual working memory, memory, aging, contralateral delay activity, event-related potentials, EEG

## Abstract

Negative affect (NA) has been related to lower working memory performance across all ages, including in older adults where it has been suggested as a marker for later cognitive impairments. However, NA-related decreases in working memory performance have not been shown in a full range of working memory paradigms or fully explored in the context of electrophysiological measures of working memory. We examined the impact of NA and related markers on an electroencephalography (EEG) marker of visual working memory (VWM) capacity, referred to as the contralateral delay activity (CDA). This study analyzed data collected from 48 cognitively intact, mildly stressed older adults (50–74 years old) who completed a VWM change-detection task to elicit the CDA, as well as self-rated measures of affect, stress, neuroticism and depression. Regression analyses revealed significant CDA amplitude effects with NA across task conditions. These results indicate a reduction in a physiological measure of VWM capacity in high-NA participants. These results are of interest as NA has been associated with a greater risk for worse cognitive function, detrimental health outcomes and reduced quality of life in older adults. This research adds to our understanding of how NA impacts older adults and gives a potential biomarker for successful intervention outcomes.

## Introduction

Visual working memory (VWM), also referred to as the visuospatial sketchpad (Baddeley, 2003), is a memory system that establishes the ability to attend to and work with visual information over a time span of seconds. This system requires active attentional engagement and contents held in memory are subject to executive manipulation. In recent years, event related potential (ERP) techniques have led to advancements in our understanding of VWM function in humans. An ERP with promising results for working memory exploration, termed the contralateral delay activity (CDA), was found to be reflective of discrete item capacity (Vogel et al., 2005; Drew et al., 2006; Ikkai et al., 2010) during working memory item retention. This ERP may be evoked, along with a measure of behavioral performance (K-score), using a computerized change detection task with varying levels of target and distractor items. The sensitivity of this component to the number of items in VWM makes it a useful marker for experiments pertaining to memory effects (see Luria et al., 2016 for a review of these recent CDA studies).

Negative affect (NA) and related conditions are known to have detrimental effects on working memory (Mitchell and Phillips, 2007; Fajkowska and Eysenck, 2009; Kremen et al., 2012). NA is a measurement of a person’s disposition to adverse emotional states. Since the original validation of the Positive and Negative Affect Schedule (PANAS) in the late 1980s, NA has been highly correlated with facets of stress, anxiety, and depression (Watson et al., 1988a; Mroczek and Almeida, 2004). Previous research has shown NA to be associated with worse working memory when measured within-person over days in an n-back paradigm (Brose et al., 2012) and between-person in a color recall task via induced NA (Spachtholz et al., 2014). Chronic psychological stress and depression, aspects of NA, also contributes to lower cognitive function, an effect which may increase with age (Lupien et al., 2005; DeRubeis et al., 2008; Stawski et al., 2010; Oken et al., 2011). In addition to cognitive changes, NA may be associated with risks for aging or resiliency to stressors in older adults (Montpetit et al., 2010; Wilkes et al., 2013). Greater NA and depression have been associated with increased subjective memory impairments in older adults (Dux et al., 2008) and may predict development of mild cognitive impairments and dementia (Wilson et al., 2003, 2007; Ownby et al., 2006). How NA and these other measures interact in non-clinical groups may shed light on a mechanism underlying observable memory decrements and clinical outcomes. Despite the breadth of previous research on NA and working memory, there is currently nothing to suggest how NA may directly impact VWM in older adults, especially using electroencephalographic (EEG) markers.

When compared to younger cohorts, older adults appear to rely on different aspects of early sensory processing, attentional filtering, and brain regions when engaging their working memory systems (Grady, 2012; Turner and Spreng, 2012). Work done by Gazzaley et al. (2008) and Jost et al. (2011) suggests that, as an individual ages, they become less efficient at selecting and encoding relevant target information into working memory, although these differences become less pronounced after the initial encoding stage. Relatedly, with aging there is some increased frontal activation that may compensate for declines in early sensory processing (Grady, 2012). These studies have also demonstrated a link between age-related VWM deficits and qualitative differences in the CDA. Specifically, detrimental aging effects were found to be most prominent in earlier time intervals of the CDA, which is thought to represent the selection and encoding of visual information (Yamaguchi et al., 1995; Gazzaley et al., 2008; Jost et al., 2011; Störmer et al., 2013). Schwarzkopp et al. (2016) explored these age-related differences alongside filtering efficiency (FE), the ability to filter out irrelevant stimuli whilst maintaining the relevant, and suggested that older adults rely on the earlier CDA interval to filter out irrelevant information; whereas, younger adults did so much earlier in time via sensory selection processes. These results indicate that there may be a fundamental dissociation between functional processes and overall performance in working memory as people age.

Recent studies on EEG measures of VWM have evaluated the effects of various mood and anxiety disorders in young populations (Klein and Boals, 2001; Schoofs et al., 2008; Owens et al., 2012; Wang et al., 2013; Qi et al., 2014). In studies using CDA, it was found that individuals with high levels of mood and anxiety disorders had either decreased storage capacity or a decreased ability to filter out irrelevant information (e.g., FE). To our knowledge, there has never been an analysis of the relationship between the CDA, FE and NA. Considering the impactful role of NA on assorted measures of WM in older adults, the CDA stands out as a promising target for exploration in examinations of NA and WM. Additionally, similar models for studying mood and anxiety disorders in VWM have already been used as an assessment of progress in intervention and biofeedback (Schoofs et al., 2008; Arend and Zimmer, 2012; Wang et al., 2013). In this group of mildly stressed older adults, higher NA should be associated with decreased onboard capacity and FE as measured by EEG (CDA) and behavioral measures (K-score).

## Materials and Methods

### Participants

Forty-eight participants were a consecutive subset out of 134 people who were recruited from the greater metropolitan area of Portland, Oregon for a mindfulness meditation intervention study (Oken et al., 2017). This study was carried out in accordance with the recommendations of the OHSU Institutional Review Board (IRB) with written informed consent from all subjects. All subjects gave written informed consent in accordance with the Declaration of Helsinki. The protocol was approved by the IRB. The data described in this article were collected at the baseline visit, prior to randomization or intervention, from a subset of participants who performed the experimental VWM task. Participant demographic information is presented in Table 1. Participants for these analyses were between the ages of 50–74 years of age. Only one participant identified as Hispanic, all others identified as Caucasian. Exclusion criteria included untreated depression as determined by interview and a score greater than five on the 15-item Geriatric Depression Scale (Yesavage and Sheikh, 1986; Lesher and Berryhill, 1994), cognitive impairments as evidenced by a Modified Telephone Interview for Cognitive Status score less than 32 (Brandt et al., 1988; Knopman et al., 2010), and the presence of any significant EEG-altering medications (e.g., stimulants and narcotics). In the initial phone screening, participants were required to have at least mild levels of self-rated stress, indicated by a Perceived Stress Scale (PSS) score (Cohen et al., 1983) greater than 9. After providing consent, but before being randomized, participants provided self-report measures of stress, neuroticism, mindfulness, energy, handedness and negative mood (see below). Questionnaires relating to the intervention or pertaining to aspects other than NA were not used in the current analysis.

**Table 1:** Participant demographic information.

	Participants (*N* = 48)
Age (years): mean ± SD (range)	58.4 ± 6.6 (50–74)
Gender:
Female	40
Male	8
Education: mean ± SD (range)	16.9 ± 2.4 (12–23)

*Demographic information reveals a highly-educated, predominantly Caucasian female sample. All participants were adults over age 50 years (mean = 58.4)*.

### Visual Working Memory Task

**Figure 1:**
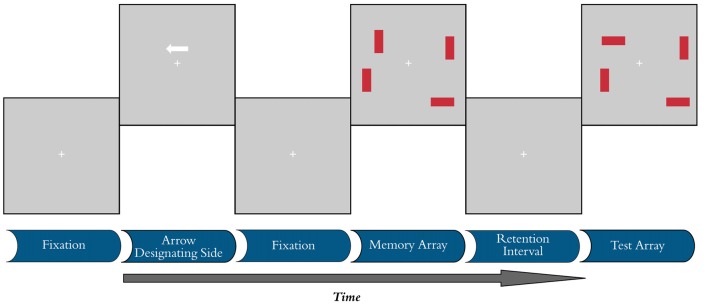
Visual working memory (VWM) task. Each trial began with a 100 ms fixation period, followed by a cue designating the target side for 200 ms. Another fixation without the cue was then randomly presented for 300–500 ms before presentation of the memory array for 200 ms. The retention interval lasted for 900 ms, after which the test array was presented. Condition C20 is shown, where there are two rectangles of target color with no distractors present. The correct participant response in the illustrated test array is “different.”

A computerized change detection task was administered to examine the CDA and behavioral measures of VWM (Vogel et al., 2005). Participants were asked to remember the orientations of a set of rectangles presented on the screen and discriminate the presence or absence of subsequent changes in the tilts of these stimuli (Figure 1). Individuals were instructed to maintain fixation on a central crosshair and to refrain from moving their eyes or blinking during trials. All stimuli presented during this task were rendered against a gray background using Presentation software (Version 16.3, Neurobehavioral Systems) on a 16″ × 12″ cathode ray tube monitor cycling at 60 Hz. Participants were seated on a high back chair at a viewing distance of 80 cm, although head position was not constrained. The task was divided into five blocks, each with 120 individual trials (40 of each test condition), with less than 1 min between blocks. Early protocol testing indicated older participants had disproportionate difficulty with the timing parameters of the original CDA paradigm, so adjustments were made to improve accuracy by adding a 100 ms fixation not used in previous versions to increase memory array presentation time to 200 ms. Similar adjustments have been made in other studies (Jost et al., 2011). The task lasted approximately 40 min, depending on the average response times of individual participants.

Each trial began with a 100 ms blank fixation period, during which participants were asked to fixate on a white cross subtending 1.90° visual angle with a stroke width of 0.11° at the center of the screen. This period was followed by the appearance of a white arrow cue 1.40° center-to-center distance directly above fixation for 200 ms. The cue subtended 1.68° in length and designated which side of the screen would contain the target array. A second blank fixation interval followed the cue and was presented for a pseudo-random duration (300–500 ms) before the appearance of the memory array for 200 ms. Each memory array consisted of either two or four 2.24° by 0.56° target red rectangular objects, with either 0 or 2 blue distractors of identical dimensions. Test conditions in this study are described as C20, C22 and C40, where the first numerical digit indicates the number of targets present and the second digit indicates the number of distractors present on screen. Thus, the C22 and C40 conditions both present four objects on the display, but only two objects need to be stored in VWM in the C22 condition if one adequately filters out the irrelevant (distractor) objects. Figure 1 demonstrates the C20 condition (2 items, no distractors), where the correct response is “different”. All rectangles were constrained to appear with 0, 45, 90, or 135° tilts at locations 3.50° or 6.99° left and right of fixation and 3.50° above or below the horizontal meridian. Individual stimulus locations within this framework were subject to pseudo-random position jitters of ±0, 0.22, 0.34, or 0.45° and ±0, 1.12, 1.68, or 2.24° along the x- and y-axes of the display, respectively. Display configurations were balanced within each block of the task. A 900 ms retention interval followed the memory array before the presentation of the test array. At test, the fixation cross turned from white to green and participants were instructed to indicate with a button press if any of the stimuli in the target array had changed orientation from the previous memory array. Although no absolute response time limit was imposed, observers were encouraged to respond within 1 s of the onset of the test array. If no response was recorded, the fixation cross turned from green to yellow to red after 1 and 2 s, respectively. Responses recorded after 2 s were counted as “misses” and excluded from behavioral calculations of working memory capacity (K). After responding, participants waited through a 2 s inter-trial interval before the start of the next trial. Working memory capacity (K) was determined using hit rate (H), false alarm rate (F), and the number of visual object stimuli (S; 2 or 4). The formula for calculating the VWM capacity is K = S(H − F) (Cowan, 2001). Capacity was measured for all three test conditions and averaged across the five blocks. FE was calculated using the proposed method from Vogel et al. (2005), where FE = (C40 − C22)/(C40 − C20).

### Self-Report Measures

Positive and Negative Affect Schedule (PANAS) is a 20-item self-report measure that quantifies positive (PANAS-pos) and negative (PANAS-neg) affect (Watson et al., 1988b). Although these components can be examined independently of one another, this study only uses the PANAS-neg component to assess NA. Higher scores on PANAS-neg indicate higher negative disposition or affect. This measure has been found to be a highly reliable with Cronbach’s alpha ranging from 0.84 to 0.87 (Watson et al., 1988c; Crawford and Henry, 2004). The Center for Epidemiologic Studies Depression (CES-D) 20-item, 4-points Likert scale is a screening for depression and its symptoms within the past week (*α* = 0.88–0.91; Radloff, 1977). A high score on the CES-D is indicative of depressive symptoms or perceptions. Neuroticism (NEO) has been considered a stable personality trait and was acquired with the 60-item NEO-FFI, a 5-point Likert scale with five factors (McCrae and Costa, 2010). The NEO factor consists of 12 items (*α* = 0.68). Higher scores on the NEO are indicative of greater neuroticism. The PSS (*α* = 0.76) is a 10-item scale that measures how situations within the past week are evaluated as stressful (Cohen et al., 1983; Lee, 2012) with greater scores indicative of more stress. Handedness was assessed with the short form of the Edinburgh Handedness inventory (Veale, 2014).

### EEG Recording

EEG data were collected using a 32-channel ActiveTwo system comprised of 32 active-sensor Ag-AgCl electrodes (BioSemi, Amsterdam, Netherlands) placed at Modified Combinatorial Nomenclature (MCN) sites AF3/4, F7/8, F3/4, FC1/2, FC5/6, T7/8, C3/4, CP1/2, CP5/6, P7/8, P3/4, P03/4, O1/2, Oz, Fz, Cz and Pz. Additionally, recordings were taken from both the left and right mastoid, while electrooculography was recorded from two electrodes placed approximately 2 cm above and below the left and right external canthi, respectively. Electrodes were referenced online to common mode sense and driven right leg electrodes, situated halfway between Cz and C3/4, respectively, and later re-referenced offline to the average of the two mastoid electrodes. Offset values were determined prior to recording and constrained to remain at a resting state of ≤25 mV to reflect adequate electrode-scalp connectivity. All EEG data were acquired using BioSemi ActiView software and a BioSemi ActiveTwo converter box with a sampling rate of 1024 Hz, DC coupled amplification, and analog-to-digital resolution of 24 bits. Participants were told to remain still, keep muscles unclenched, and remain attentive during the experiment. During the session, data quality was actively monitored using the ActiView interface in a separate room by a member of the study team. Data were analyzed offline using BrainVision Analyzer (Version 2.0.1.3931, Professional Edition) software and using a 70 Hz low pass filter, a 0.1 Hz high pass filter and a 60 Hz notch filter. Only EEG data from trials with correct responses were included in the analysis. All ERPs discussed henceforth were derived from lateralized averages across electrode sites CP5/6, P3/4, P7/8, P03/4 and O1/2, which is consistent with previous noted CDA literature and the posterior distribution of the potential (see “Results” section for topographical analyses).

### EEG Data Processing and CDA Analysis

Independent Component Analysis was used to remove eye-related electrical activity. Artifact rejection was employed to remove sections of data contaminated by muscle activity, electrode malfunction, or abrupt head movements. The criteria for semi-automatic artifact rejections were as follows: maximal allowed voltage step of 50 μV/ms; maximal allowed absolute difference 125 μV within interval length of 50 ms; maximum EEG amplitudes allowed was ±75 μV and the lowest allowed EEG amplitude was 0.5 μV within interval length of 100 ms. EEG activity from 200 ms before to 200 ms after artifact events was eliminated. Using these criteria in addition to the electrooculography channel, eye saccades and other ocular artifacts were identified and removed. Participants were not included if more than 50% of the EEG was removed for any single condition (C20, C22 and C40). These thresholds were implemented in order to ensure adequate resolution of each condition. No incorrect trials were used during segmentation or included in the final calculation of the VWM components in this study. After independent component analysis and artifact rejection, channels were separated into contralateral and ipsilateral sides relative to the target array and averaged within each condition. This average was then exported as a signed mean activity measure, using the interval 200 ms preceding the VWM array presentation for baseline correction. The mean activity between 300 ms and 1100 ms after the onset of the target memory array was exported separately for both contra and ipsilateral sides; the difference of these two areas representing the CDA.

### Statistical Analysis

Regression analyses were performed using Stata (Version 11.1) statistical software. All data were tested for normality using the Shapiro–Francia test. When normality was not observed non-parametric Spearman correlations were used to test hypotheses. Regressions were all tested for an interaction with age and it was included in the analyses. These analyses were exploratory and no correction was made for multiple comparisons. In general, when the results from the three stimulus conditions were consistent, this provided validation of statistically significant findings.

## Results

We recruited and analyzed data from 48 participants (see Table 1 for descriptive measures). Only 7 of the 48 participants reported left hand preference; a two-tailed *t*-test comparing left and right-handed groups revealed no significant (*p* > 0.75) differences in any of the three CDA conditions analyzed below in which handedness might impact results. Given this observation, all data were pooled and analyses were done on the full sample. Descriptive summaries of our self-report measures are outlined in Table 2. All variables were normally distributed as indicated by *p* > 0.05 on the Shapiro–Francia test.

**Table 2:** Summary of self-report measures.

Scale	Mean (Std.Dev)	Range
NA	20.19 (6.36)	11–36
PSS	17.90 (5.80)	4–29
*NEO—Neuroticism*	27.38 (8.47)	13–45
CES-D	17.17 (9.29)	1–43

*Mean, standard deviation and range for the self-report questionnaires*.

### Contralateral Delay Activity (CDA)

**Figure 2:**
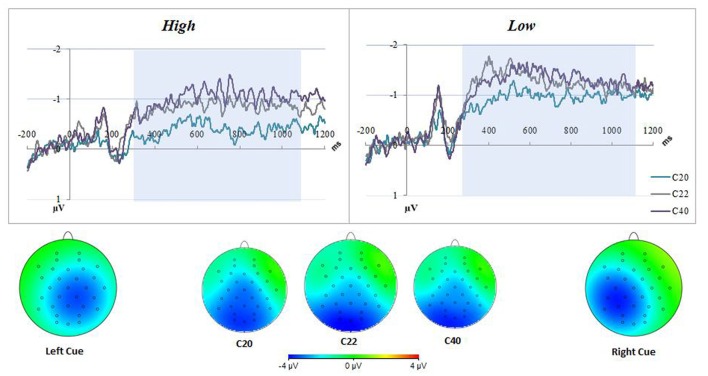
Negative affect (NA) difference waveforms for all conditions. The top figure represents the difference waveform (contra-minus-ipsilateral) for all conditions, for two groups of participants generated by median (score of 19) low-high split of positive and negative affect schedule (PANAS)-neg scores. There were observable set-size effects in both groups; having C22 closer to C40 and C20 lowest in amplitude. The low PANAS-neg figure also demonstrates an important dissociation between groups, namely the higher amplitude contralateral delay activity (CDA) overall. The CDA interval used for analyses was highlighted in blue. The bottom of the figure displays heatmaps for both all conditions, averaged across all participants in the CDA interval, and separated by Left/Right Cue. The averaged heatmaps in the center indicate a posterior dominant potential across conditions. The Left/Right Cue heatmaps demonstrate a lateralized potential with some diffusion across hemispheres.

**Figure 3:**
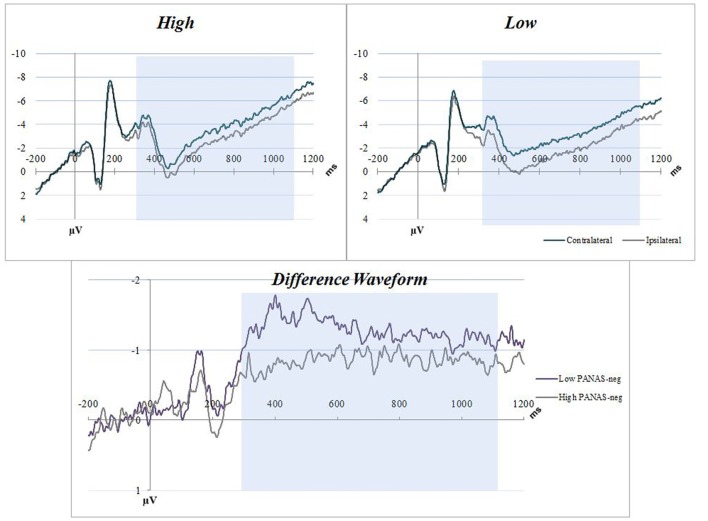
NA C22 averaged waveforms. The graphs have separated the sample into low-high PANAS-neg scores by median split. Blue boxes indicate the CDA interval (300–1100 ms). Top figures are event related potential (ERP) plots without subtraction for Contralateral and Ipsilateral, split between high (top left) and low (top right) NA. The bottom figure is a difference waveform comparing the high and low PANAS-neg participants for the C22 condition.

Distribution and summary measures of the CDA are reported in Table 3. In all conditions, mean activity of the CDA correlated significantly with NA (*p* < 0.05). NEO, CES-D and PSS were only significant for the C20 condition. Beta coefficients, *R*^2^, and significance levels are reported in Table 4. Surprisingly, although NA was significantly related to CDA activity across all conditions, CES-D was the most significant predictor of CDA amplitude in the C20 condition, followed by PSS. A median split between the high and low NA groups in ERP and difference waveforms demonstrate the amplitude differences from our regressions (see Figures 2, 3), indicating lower amplitude in the CDA interval with higher NA. As expected for this experiment, there were observable set-size effects: visually participants had more activity in the C22 and C40 conditions (four on-screen items) than in the C20 condition, which only presented two items (see Figure 2). The heatmaps present in the same figure depict the CDA as a posteriorly dominate and lateralized potential.

**Table 3:** Summary of contralateral delay activity (CDA) amplitude measures.

CDA task conditions	All participants Mean (Std.Dev) *N* = 48	Range	High NA Mean (Std.Dev) *N* = 23	Low NA Mean (Std.Dev) *N* = 25
C20(uV)	−0.667 (0.844)	−2.61 to +1.45	−0.952 (0.812)	−0.321 (0.75)
C22(uV)	−1.13 (0.889)	−3.22 to +0.714	−0.860 (0.724)	−1.40 (0.961)
C40(uV)	−1.18 (0.729)	−2.61 to +0.225	−0.951 (0.627)	−1.40 (0.763)
FE	0.442 (4.70)	−19.04 to 15.21	−0.876 (4.73)	1.54 (4.55)

*Mean, standard deviation and range for the Contralateral Delay Activity and Filtering Efficiency. Highlighted columns indicate mean and stand deviation values for two groups of participants generated by median (score of 19) low-high split of PANAS-neg scores (labeled High NA and Low NA)*.

**Table 4:** Association of CDA and Negative affect (NA) measures.

Self-report scales	C20	C22	C40	FE
NA	0.374** (0.143)	0.344* (0.119)	0.396** (0.157)	−0.092
PSS	0.389** (0.151)	0.149 (0.02)	0.239 (0.056)	−0.33*
CES-D	0.431*** (0.191)	0.285 (0.089)	0.247 (0.065)	−0.152
NEO—Neuroticism	0.323* (0.100)	0.191 (0.036)	0.247 (0.059)	0.123

Regression analysis of CDA amplitude demonstrated it was significantly associated with NA across all conditions. All other measures were significant for the C20 condition, but not for C22 or C40 conditions. Reported numbers are Beta Coefficients with asterisks indicating the following for p-values: ***p < 0.005, **p < 0.01, *p < 0.05. The parentheses indicate R^2^ values. FE was not normally distributed, even after transformation, therefore Spearman rho is given with same asterisk meaning as for the regressions.

### Filtering Efficiency (FE)

Distribution and summary measures of FE are reported in Table 3. Of note, there were nine values less than 0 and 16 greater than 1. Although Vogel et al. (2005) mentions FE values outside of the 0–1 range were possible, especially in cases when activity assumptions are violated (C20 < C22 < C40), such values were not observed in his college-age population with more trials per participant. To analyze FE further in any meaningful way, since FE is supposed to represent a 0–1 efficiency, we set all values less than 0 to be equal to 0 and all values greater than 1 to equal 1. This measure was not normally distributed before or after this re-calculation, therefore non-parametric Spearman correlations were performed (see Table 4). PSS was significantly associated with FE (*p* = 0.02), but not with the other measures (*p* > 0.05).

### Capacity-Performance (K-Score)

The K-Score did not significantly correlate with any of the self-report measures (see Table 5 for K-Score summary measures). There was a significant regression model that incorporated C22, age and K22, *F*_(2,44)_ = 3.24, *p* < 0.05, *R*^2^ = 0.128, with age contributing negatively (*β* = −0.249) and C22 activity contributing positively (*β* = 0.255) to the K-score. Similar regressions for K-score in the other two CDA conditions were insignificant (*p* > 0.05). Using Spearman correlations, there were no significant associations of FE with any of the condition K values (*p* > 0.4).

**Table 5:** Summary of K-score measures.

K-score task condition	Mean (Std.Dev)	Range
K20	1.6 (0.22)	0.75–2
K22	1.4 (0.34)	0.45–1.9
K40	1.8 (0.52)	0.44–3.1

*Mean, standard deviation and range for the performance measure (K-Score)*.

## Discussion

This study examined an ERP component of VWM in relation to NA in a sample of older adults. Regression analyses indicate significant negative correlations between NA and these VWM capacity measures. This relationship (see Figures 2, 3) demonstrates the degree to which these CDA amplitudes are impacted by higher NA. VWM performance (K-score) was only significantly related to CDA and age in the filtering condition (C22), inherently the most difficult of the three conditions. It is our interpretation that NA may contribute to decreases in onboard storage of relevant information maintained in VWM (CDA amplitude). Our hypothesis that NA would negatively impact FE was not supported by our data.

Though we observed no robust relationship between NA and K-score in the current study, other researchers have produced contrary findings. In a study on VWM and NA by Spachtholz et al. (2014), reduced behavioral VWM capacity was found to accompany higher NA. However, that study was done in a younger group and with experimentally induced NA, which might explain why our results were not able to replicate such a link with NA and K-score. In analyses done on older adults, non-induced NA is highly correlated with subjective memory complaints, an area of prolific research in cognitive aging fields for its potential utility as an early marker for cognitive declines (Dux et al., 2008; Wilkes et al., 2013). It is possible that these underlying CDA capacity measures are sensitive to smaller individual changes in cognitive processing that do not manifest as performance deficits until later. The interaction between K-score and CDA amplitude has been previously reported in both young and old sample groups (Jost et al., 2011; Sander et al., 2011; Owens et al., 2012), something our study was not able to replicate. However, there is evidence replicating our dissociation between K-score and CDA measures (Ko et al., 2014; Qi et al., 2014). Of particular interest, in a study conducted by Newsome et al. (2013) on the impact of mild cognitive impairment on the CDA and P300, researchers found no difference between healthy older and young adults for either K-score or CDA amplitude. However, they did find significantly reduced CDA amplitudes and impaired K-scores in those at-risk of cognitive impairments. It is probable that proper exclusion for cognitive impairments would help resolve disagreements on the interaction of aging, K-score and the CDA. Additionally, Sander et al. (2011) posited that CDA and K-score may be dissociated in cases where top-down control is experimentally limited, such as in cases of particularly long or short presentation times. It is conceivable that the addition of the 100 ms fixation period in our modified design may have incidentally resulted in such a scenario for our sample of older adults, although additional tests are needed to verify this possibility. Lastly, the CDA, since it is computed using only correct trials, may not be sensitive to task difficulty. However, the K-score may be compromised by alterations in task difficulty (Luria et al., 2016) which may account for some of the dissociations seen in the literature.

We observed calculated FE values outside the 0–1 range, which had been stated to be possible by Vogel et al. (2005). Studies have even opted for alternative difference calculations (Jost et al., 2011) to determine FE over the course of the CDA interval in older adults; therefore, it is unknown if this is typical for the measure in this older population. This extended range is likely related to lower signal to noise ratio of CDA amplitude measurements in older adults in general and to our use of fewer stimuli. We did observe a relationship between FE and PSS such that those with higher perceived stress had lower FE. While this suggests a negative impact of PSS on FE in addition to WM capacity, this relationship was not seen with the other NA measures. One possible explanation for these findings may be use of the full CDA time window used in our study as was done in the original Vogel et al. (2005) publication. Of interest, Jost et al. (2011) observed that older adults did not exhibit efficient filtering until a latency of approximately 600 ms. Future studies should consider exploring FE over different time courses of the CDA and with a greater number of conditions to determine potential NA relationships.

While there is no previous literature on NA and the CDA, there have been studies on highly correlated disorders such as anxiety and depression (see Brown et al., 1998 for correlations, in particular Figure 2) in younger adults. A study of young adults conducted by Qi et al. (2014) observed a reduction in working memory capacity with increases in trait anxiety, as well as differences in ERPs in participants with High Trait Anxiety (HTA), similar to the high-NA participants in this current study. Dysphoric attributes also appear to play a role in VWM performance. Owens et al. (2012) reported a decline in VWM filtering performance in depressed (or dysphoric) patients. Our study did not replicate Owens et al.’s (2012) findings with depression specifically, but this discrepancy may be a result of our exclusion of clinically untreated depression, or a narrower demographic (age, gender and education). However, these criteria were necessary for this study in order to isolate the impact of NA on VWM as compared to other highly related disorders (Brown et al., 1998). Our findings, taken with previous studies on depression, should encourage the usage of a depression scale to rule out depression or HTA as the contributing factor to underlying VWM function.

This study has several limitations which should be considered when interpreting these findings. While these results are in line with previous research on both NA and CDA, there is no younger control group with which to compare our results. NA may differentially impact other age groups, which seems likely given working memory differences with age. Additionally, this study consisted of at least mildly stressed, primarily highly-educated Caucasian females, which limits the generalizability of our results. Additionally, while other research on the CDA in older adults has noted dissociation between CDA and K-score measures on capacity, as mentioned above, it should be considered a limitation and interpreted with caution until more research addresses this issue. To confirm the preliminary findings reported here, participants in future investigations should include younger groups, an increased demographic range, and a broader range of NA.

In conclusion, our hypothesis that NA would impact onboard measures of VWM capacity was supported by our analysis; there were significant attenuations in CDA amplitude in older adults with greater NA. Our findings are of interest for older adults with high NA, a group at risk for later cognitive impairments and other disorders, and indicate a promising avenue for future research. Studies like these may help develop screenings for and methods to alleviate the working memory decrements that result from aging, increased NA, and other mood disorders.

## Author Contributions

TM processed and analyzed data, and wrote the manuscript. DK assisted in processing of data and writing of manuscript. BO oversaw the project, provided statistical analysis and assisted in writing of the manuscript.

## Conflict of Interest Statement

The authors declare that the research was conducted in the absence of any commercial or financial relationships that could be construed as a potential conflict of interest.
